# Examining Procrastination among University Students through the Lens of the Self-Regulated Learning Model

**DOI:** 10.3390/bs10120184

**Published:** 2020-12-01

**Authors:** Pierpaolo Limone, Maria Sinatra, Flavio Ceglie, Lucia Monacis

**Affiliations:** 1 Department of Humanities, University of Foggia, 71100 Foggia, Italy; pierpaolo.limone@unifg.it; 2 Department of Educational Sciences, Psychology, Communication, University of Bari, 70121 Bari, Italy; maria.sinatra@uniba.it; 3 Department of Pathological Anatomy, University of Bari, 70120 Bari, Italy; flceglie@libero.it; 4 Department of Economics, Management and Territory, University of Foggia, 71100 Foggia, Italy

**Keywords:** academic procrastination, time management, cognitive strategies, metacognitive strategies, gender differences

## Abstract

Generally considered as a prevalent occurrence in academic settings, procrastination was analyzed in association with constructs such as self-efficacy, self-esteem, anxiety, stress, and fear of failure. This study investigated the role played by self-regulated learning strategies in predicting procrastination among university students. To this purpose, the relationships of procrastination with cognitive and metacognitive learning strategies and time management were explored in the entire sample, as well as in male and female groups. Gender differences were taken into account due to the mixed results that emerged in previous studies. This cross-sectional study involved 450 university students (M = 230; F = 220; Mage = 21.08, DS = 3.25) who completed a self-reported questionnaire including a sociodemographic section, the Tuckman Procrastination Scale, the Time Management Scale, and the Metacognitive Self-Regulation and Critical Thinking Scales. Descriptive and inferential analyses were applied to the data. The main findings indicated that temporal and metacognitive components play an important role in students’ academic achievement and that, compared to females, males procrastinate more due to poor time management skills and metacognitive strategies. Practical implications were suggested to help students to overcome their dilatory behavior.

## 1. Introduction

In 1886, William James wrote in a letter to Carl Stumpf: “Nothing is so fatiguing as the eternal hanging on of an uncompleted task” [1]. James’ sentence expressed very well the sense and the psychological cost of putting something off until a later time, i.e., of procrastinating, an issue of focus of many researchers, particularly since the second half of the nineties of the twentieth century, although the term ‘procrastination’ has a longer history [2,3]. Variously described as the irrational delay of behavior [4,5,6], the delay in beginning or completing an intended course of action [7,8,9], or the voluntary delay of an intended course of action despite expecting negative consequences [3,4,5,6,7,8,9,10], according to Milgram and Tenne [11], the construct refers to a trait or a behavioral disposition of postponing or delaying performing a task or activity with no apparent reason.

Following the two main traditions of research, that is, as a stable personality trait or as a behavior closely linked to the characteristics of a given situation [12], procrastination was investigated from five perspectives, including: 1. general procrastination, referring to a dysfunctional behavior negatively associated with health, wealth, and happiness [13] as well as to difficulty in meeting deadlines within a specific time-frame; 2. academic procrastination, considered as a pervasive and permanent desire on the part of the learner to postpone academic activities, such as deferring study the night just before the exam—this form of procrastination is usually accompanied by anxiety [14,15]; 3. decisional procrastination, related to a stable maladaptive pattern of postponing a decision when faced with conflicts and choices [16,17]; 4. neurotic procrastination, referring to difficulties with building critical judgments and making well-timed life decisions [18]; 5. compulsive or dysfunctional procrastination, referring to the coexistence of both decisional and behavioral procrastination [19], where the latter is related to failing task completion, feeling guilty after a positive event, and choosing handicapping situations.

Much literature has focused on academic procrastination, defined as a tendency to delay academic tasks to the point of experiencing anxiety associated with them. The concept is derived from behavioral theories, according to which the act of delaying academic tasks is interpreted as a task-specific avoidance behavior associated with Skinner’s theory of learning and reinforcement [20,21]. Academic procrastination should be prevalent among students who have been directly or indirectly given rewards or have not been receiving enough punishment for this act of purposely delaying academic assignments [2].

An important question investigated by various researchers concerns the relationship between procrastination and students’ academic performance. Indeed, meta-analytic reviews [3,22] have confirmed that procrastination negatively affects academic performance: Academic procrastinators not only are limited in the establishment of a calendar of activities, but they also lack a self-regulatory pattern that includes learning goals and metacognitive processes [23].

In line with Ferrari’s [24] statement that procrastination should be understood as a self-regulation failure of students’ performance when placed in stressful situations, this construct was also examined while taking the self-regulated learning (SRL) perspective into account. Based on a general cognitive model of learning and information processing, and intended as ‘‘an active, constructive process whereby learners set goals for their learning and then attempt to monitor, regulate, and control their cognition, motivation, and behavior, guided and constrained by their goals and the contextual features in the environment’’ [25] (p. 453), SRL includes the cognitive, metacognitive, behavioral, motivational, and emotional/affective aspects of learning oriented toward academic achievement [26,27] and can be encapsulated in three components: (a) metacognitive strategies for planning, monitoring, and modifying cognitive processes, (b) management and control of students’ effort in classroom academic tasks, (c) and actual cognitive strategies used to learn, remember, and understand the material. Further research demonstrated that these cognitive strategies have determined an enhanced understanding of information and also promoted critical thinking, knowledge transfer, and problem-solving skills, thus leading students to higher grades [28]. The cognitive strategy of critical thinking, which refers to the activation of relevant prior content knowledge and involves a form of higher-order cognitive engagement [29], is one of the main aspects in the process of self-regulation learning, as greater levels of critical thinking are associated with greater cognitive engagement and successful performance.

Thibodeaux and colleagues [30] have highlighted that students engaging in SRL reported more metacognitive and cognitive strategies for learning. Conversely, Park and Sperling [31] demonstrated that students with low SRL tended to miss or attend class late, to delay academic tasks, and to drop out of university. Similarly, procrastinators seemed to have a deficit in regulating their cognition and metacognitive knowledge and skills [32,33,34], thus showing low levels of self-efficacy and self-esteem [35], as well as high levels of anxiety [36,37], stress, fear of failure [26], and discomfort regarding tasks [38,39,40,41].

Following Wolters’ suggestion [42] that specific factors of self-regulated learning should be explained in order to more deeply analyze students’ tendency to procrastinate, time management was also taken into account in the current study to find out its unique variance in procrastination beyond what is accounted for by cognitive and metacognitive learning strategies. In light of the lack of a common definition of time management, this study considered Claessens and colleagues’ [43] conception that the construct refers to “behaviors that aim at achieving an effective use of time while performing certain goal-directed activities” (p. 262). As such, it should be seen as a multidimensional process, which fits within the SRL framework, wherein students deliberately regulate when, where, and for how long they engage in academic tasks [44]. In this sense, it is associated with students’ use of strategies and motivational beliefs [45]. Previous studies that generally used a scale developed by Pintrich et al. [46] to measure students’ general self-beliefs about whether they manage their time well reported positive correlations of the use of time management with the use of cognitive, metacognitive, and self-regulatory strategies aimed at achieving academic success [31,47,48,49,50]. In support of the key role played by time management in academic contexts, students who received training in managing time improved their academic achievement and well-being [51,52,53]. In the current study, time management was considered while taking Weinstein’s Model of Strategic Learning (MSL) into account [54]. The model conceptualizes academic learning as strategic or autonomous, and includes three primary components, i.e., skill, will, and self-regulation, where the first refers to critical knowledge, the second to motivational and affective components of strategic learning, and the third to how students manage their strategic learning on both the global and the real-time level. The third component, used as an overall framework for the merging of the other two components, comprises managing time over weeks, months, and years on the global level, whereas on a more immediate basis, that is, during a task, over a few hours or day by day, on a real-time level [55].

In order to learn much more about the characteristic tendency to procrastinate as a function of individual differences, demographic variables, such as gender, have been taken into account as potential indicators of academic procrastination. Previous studies reported mixed findings, ranging from non-notable [56,57,58] to significant gender differences: Some authors found that males obtained higher levels of procrastination [3,59,60,61,62,63], whereas others reported that females procrastinated more frequently [64].

Aiming at further empirically contributing to the role played by self-regulated learning strategies in predicting procrastination among students, the current research sought to: 1. verify the relationships of procrastination tendency with metacognitive learning strategies and time management, and 2. provide empirical evidence on gender differences in academic settings. To this purpose, correlational and regression analyses were carried out in the total, male, and female samples in order to examine whether the metacognitive self-regulation and critical thinking turned out to be significant predictors of procrastination after taking time management into account.

## 2. Materials and Methods

### 2.1. Participants

The data presented were gathered from a sample of 450 (M = 230, F = 220) university students enrolled in Southern Italian universities (Mage = 21.08, DS = 3.25) who were enrolled in Humanistic and Educational Sciences. Participants were informed about the nature and the objectives of the research, and they gave written consent; it was made clear that participation was voluntary and that all data would remain confidential. The data were collected during a classroom lesson in the autumn semester of 2019 before the COVID-19 pandemic.

### 2.2. Ethics Statement

The study was carried out in accordance with the ethical principles of the Helsinki Declaration and the Ethics Committee of the local university (Reference No 12/2019).

### 2.3. Instruments

Participants were administered a self-report survey, including a sociodemographic scale and the following questionnaires:

(i) The Tuckman Procrastination Scale (TPS) [65]: To best fit the aim of this research, a truncated version of the scale with sixteen items was applied. Participants were required to rate them on a four-point Likert Scale ranging from 1 (strongly disagree) to 4 (strongly agree). This scale takes into account two types of procrastination: general explanation of procrastination (for example, “When I have a deadline, I wait until the last minute”) and likelihood to avoid difficult or unpleasant tasks (for example, “When something’s too tough to tackle, I believe in postponing it”). Tendency of procrastination was measured by the summated score of all 16 items, ranging from 16 to 64. The higher the score, the higher the procrastination tendency. In the current study, the Cronbach alpha reliability coefficient was good (0.86).
(ii) The Time Management (TM) subscale of the Learning and Study Strategies Inventory (LASSI) [66] was used to measure the level of a student’s time management qualifications. A high score indicates a good understanding of how to organize the available time and resources, whereas low scores mean that students need to learn about how to create a schedule and how to deal with distractions and procrastination. The subscale includes eight items rated on a five-point Likert scale (from 1 = not at all typical of me to 5 = very typical of me). Sample items are: “I find it hard to stick to a study schedule”; “I set aside more time to study the subjects that are difficult for me.” The total score was obtained by summarizing the answers given to the items. In the current study, the reliability coefficient was found to be acceptable (Cronbach’s alpha = 0.69).
(iii) The Metacognitive Self-Regulation (MSR) and Critical Thinking (CT) subscales of the learning strategies section of the Motivated Strategies for Learning Questionnaire (MSLQ) [46] were applied to evaluate the use of metacognition and self-regulation strategies, as well as critical thinking. The MSR subscale, focused on the control and self-regulation aspects of metacognition and not on the knowledge component, consists of twelve items scored on a five-point Likert scale (from 1 = not at all true of me to 5 = very true of me). Sample items are: “When reading for a course, I make up questions to help focus my reading”; “I try to change the way I study in order to fit the course requirements and the lecturer’s teaching style.” The CT refers to the degree to which students report applying previously acquired knowledge to new situations in order to solve problems, reach decisions, or make critical evaluations with respect to standards of excellence, that is, the scale assesses higher-order thinking skills [67]. The five items included in the scale are rated on a five-point Likert scale (from 1 = not at all true of me to 5 = very true of me). Sample items are: “Whenever I read or hear an opinion or conclusion in a course, I think about possible alternatives”; “I often find myself questioning things I hear or read in a course to decide if I find them convincing.” The total score for each scale was obtained by calculating the average of the answers given to the items. In the current study, both scales showed adequate levels of reliability (Cronbach’s alpha = 0.69 for CT; 0.77 for MSR).

In this study, forward translation of all questionnaires of the Tuckman Procrastination Scale, from English into Italian, was performed by an English native speaker. The discrepancies existing in the Italian and in the back-translations were then discussed with the authors until consensus was reached.

## 3. Results

Table 1 reports the data emerging from descriptive analyses with respect to the total sample and the gender subsamples. In general, a gender effect emerged only on the critical thinking score, t (448) = 1.065, *p* = 0.03. Females obtained higher mean values compared to males. Moreover, the data showed that, even if there were no other gender effects, females obtained higher mean scores in metacognitive strategies and lower mean scores in time management and procrastination. Zero-order correlations were carried out among the variables of interest in the total sample and in males and females. In the total sample, the findings showed that: 1. Procrastination was negatively correlated with time management and metacognitive self-regulation, and was unrelated with critical thinking; 2. time management was positively correlated with metacognitive self-regulation and critical thinking. The first correlation turned out to be stronger than the second. 3. Metacognitive self-regulation was found to be positively correlated with critical thinking (Table 1). In females and males, the findings generally confirmed the above-mentioned associations, although the negative association between procrastination and critical thinking turned out to be significant.

Regression analysis was performed using the forward method to analyze whether and to what extent cognitive learning strategies may negatively predict the tendency toward procrastination (Table 2). The order of variables was based on the obtained correlations: Step 1 included time management, and Step 2 included metacognitive self-regulation and critical thinking. In the total sample, the beta values of time management and metacognitive self-regulation showed a negative influence on procrastination, whereas the beta value of critical thinking was not found to be significant. In males, the data indicated that time management and metacognitive self-regulation were negative predictors of procrastination, whereas critical thinking showed a considerable trend toward significance (*p* = 0.057). In females, an unexpected result emerged in that time management that was no longer a significant predictor of procrastination. On the other hand, cognitive and metacognitive learning strategies were confirmed as significant predictors.

## 4. Discussion

Authors should discuss the results and how they can be interpreted from the perspective of previous studies and of the working hypotheses. The findings and their implications should be discussed in the broadest context possible. Future research directions may also be highlighted. In order to shed more light on the nature of relationships between procrastination and self-regulated learning in academic settings, this study analyzed whether cognitive and metacognitive learning strategies together with time management may predict the tendency to procrastinate. The data generally confirmed the idea that procrastination represented failure in regulating students’ behavior. Indeed, the results from the correlations highlighted that the three constructs were negatively associated with procrastination and that critical thinking obtained the lowest correlation coefficient. As expected, the dilatory behavior with respect to performing tasks was strongly associated with students’ low capability of planning their study and regulating their cognitive learning strategies. Moreover, a careful inspection revealed that the use of time and the regulation of metacognitive processes seemed to have the same strength in the negative association with procrastination tendency. That is, either knowing how to manage time together with the lack of the regulation of metacognitive processes or lacking in managing time together with knowing how to regulate metacognitive processes may lead students to procrastinate. These annotations supported the consistency of our findings with previous studies, underlining the important role played by temporal and metacognitive components in students’ academic achievement [31,32,45,50].

Following the line of research aimed at analyzing gender differences in procrastination in relation to psychological constructs, such as personality traits [58], academic satisfaction [60], self-efficacy, and self-esteem [35], the current study provided evidence for significant gender differences on the basis of the theoretical frameworks of self-regulated learning and strategic learning.

Compared to females, males showed a deficit in time management, operationalized as self-regulation, i.e., the third component of Weinstein’s model (MSL). Moreover, the male tendency to show low levels of metacognitive learning strategies appeared as another key factor in predicting dilatory behaviors in academic learning. These findings corroborated Wolters’ [42] hypothesis that time management should be considered one of the key determinants of academic procrastination. In addition, time management may be treated as a link between the SRL and MSL, as the self-regulation failure of metacognitive strategies is strictly associated with students’ low capability of planning, monitoring, and evaluating their academic tasks. By contrast, compared to males, females showed a low tendency to regulate cognitive and metacognitive processes, thus determining academic procrastination. As for the construct of time management, it did not turn out to be significant in postponing academic tasks. Therefore, these findings were consistent with those of prior investigations showing that males procrastinate more than females in academic settings [3,59,60,61,62,63].

Practically, the findings highlighted that being a self-regulated learner may reduce dilatory behaviors and that gender differences should be taken into account. In order to manage academic procrastination, educators could provide specific and targeted programs dealing with how to organize time and resources and how to improve cognitive and metacognitive skills aimed at solving problems, reaching decisions, or making critical evaluations [68]. Such target programs could improve not only students’ academic life, but also their general well-being [69,70]. This improvement is of particular importance for the inclusion of students with SEN (special educational needs), for example, students with neurodevelopmental disorders (learning disabilities, ADHD, ASD).

Although this study yielded significant findings, some limitations should be considered. First, given the cross-sectional design, the research dealt with the associations between self-regulated learning and academic procrastination, and not with causes and effects. Future investigations should carry out experimental designs to examine whether academic procrastination is caused by poor self-regulated learning strategies. Second, the self-reported measures used in this study can suffer from biases, thus affecting the results. Finally, it might be useful to explore the role of environmental and contextual factors in the manifestation of non-functional behaviors.

## Figures and Tables

**Table 1:** Descriptive statistics and correlations (N = 450).

Total Sample (N = 450)	Min–Max	M (SD)	1	2	3
1. TPS	16–64	33.20 (8.79)	-		
2. TM	15–40	29.74 (3.93)	−0.586 **	-	
3. MSR	1.58–5.00	3.81 (0.54)	−0.516 **	0.556 **	-
4. CT	1.25–5.00	3.42 (0.63)	−0.098	0.234 **	0.590 **
Males (n = 230)					
1. TPS	16–64	33.30 (8.87)	-		
2. TM	15–38	30.52 (3.67)	−0.595 **	-	
3. MSR	1.58–4.92	3.69 (0.55)	−0.520 **	0.545 **	-
4. CT	1.20–5.00	3.40 (0.62)	−0.128 *	0.224 **	0.593 **
Females (n = 220)					
1. TPS	16–63	32.98 (8.75)	-		
2. TM	15–40	28.53 (4.03)	−0.579 **	-	
3. MSR	1.67–5.00	3.92 (0.54)	−0.499 **	0.549 **	-
4. CT	1.80–5.00	4.02 (0.75)	−0.209 **	0.231 **	0.591 **

** *p* < 0.01; * *p* < 0.05; TPS = Procrastination, TM = Time Management, MSR = Metacognitive Self-Regulation, CT = Critical Thinking.

**Table 2:** Regression analysis in the total sample and in males and females.

Model for Total Sample		β	Sig.
1	TM	−0.586	0.000
2	TM	−0.433	0.000
	MSR	−0.265	0.000
	CT	−0.010	0.120
ΔR^2^ = 0.022, *p* < 0.05			
Model for male sample			
1	TM	−0.596	0.000
2	TM	−0.413	0.000
	MSR	−0.271	0.000
	CT	−0.120	0.057
ΔR^2^ = 0.010, *p* < 0.05			
Model for female sample			
1	TM	−0.579	0.000
2	TM	−0.109	0.170
	MSR	−0.290	0.000
	CT	−0.150	0.020
ΔR^2^ = 0.009, *p* < 0.05			
